# Supplementary material for: Factor-Dependent Internal Ribosome Entry Site and -1 Programmed Frameshifting Signal in the Bemisia-Associated Dicistrovirus 2
Source: Viruses. 2024 Apr 28;16(5):695. doi: 10.3390/v16050695 (PMC11125867; doi:10.3390/v16050695)

SUPPLEMENTAL FIGURE LEGEND

Figure S1: Predicted secondary model of BaDV-2 IGR IRES. A) Specific nucleotides were mutated to validate the predicted secondary structure of BaDV-2 IGR IRES. B) The luciferase activities were measured through luciferase assays for each mutation via bicistronic reporter RNA assays in Sf-21 extracts. A one-way ANOVA statistical test was used to determine the p value and thus the significance levels. Groups were normalized to bicistronic RNAs containing WT BaDV-2. *p<0.05, **p<0.01. "n.s" denotes the difference is not significant between the experimental groups and WT (p > 0.05). Shown are the averages from at least three independent experiments ± standard deviation.

Figure S2: BaDV-2 IGR IRES activity in mock-infected and CrPV-infected cells. Bicistronic reporters were transfected into S2 cells followed by mock infection or CrPV infection (MOI = 20). Cells were collected 6 hours post transfection. Bicistronic RNAs tested contain the wild-type CrPV, mutant CrPV (CC6214-5 to GG to disrupt PKI basepairing), wild-type BaDV-2 full-length IGR IRES (WT BaDV-2), BaDV-2 3' DEL 5 and BaDV "minimal" IRES (double deletion at both 3' and 5' end. A) Renilla luciferase activities and B) ratio of Renilla/Firefly luciferase activities were measured and normalized to wild-type CrPV with mock infection. A paired t-test was used to determine the p value and thus the significance levels. * represents p<0.05, ** represents p<0.01. "n.s" denotes the difference is not significant between the control groups and the experimental groups (p > 0.05). Shown are the averages from at least three independent experiments ± standard deviation.

Figure S3: SHAPE reactivity of BaDV-2 IRES. In vitro transcribed RNA was analyzed for SHAPE reactivity. Denatured vs folded RNA in the presence or absence of NMIA were analyzed by amplicon next-gen sequencing (UBC Sequencing and Bioinformatics Consortium). Relative

SHAPE reactivities for each nucleotide are depicted with colors indicated to the bottom right and are from three independent experiments.

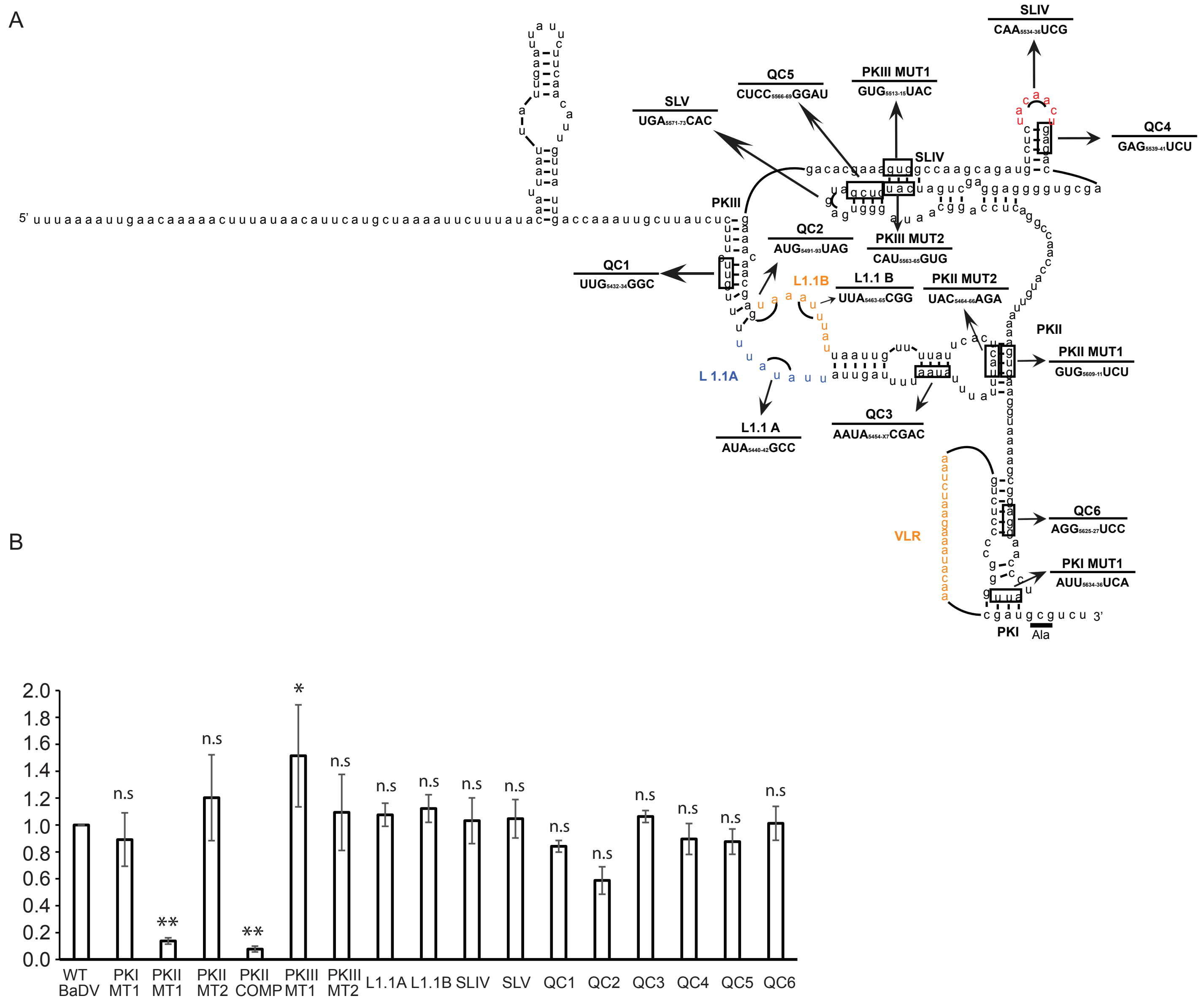

A
SLIV
CAA5534-36UCG
PKIII MUT1
GUG5513-15UAC
QC5
CUCC5566-69GGAU
SLV
UGA5571-73CAC
QC4
GAG5539-41UCU
SLIV
PKIII
5' uuuaaaaauugaacaaaacuuuauaacauucaugcuaaaauucuuuuauac-gaccaaauugcuuaucuc
QC2
AUG5491-93UAG
PKIII MUT2
CAU5563-65GUG
QC1
UUG5432-34GGC
L1.1B
L1.1 B
UUA5463-65CGG
PKII MUT2
UAC5464-66AGA
PKII
PKII MUT1
GUG5609-11UCU
L 1.1A
L1.1 A
AUA5440-42GCC
QC3
AAUA5454-X7CGAC
VLR
QC6
AGG5625-27UCC
PKI MUT1
AUU5634-36UCA
PKI
Ala
3'
B
FLuc/RLuc
2.0
1.8
1.6
1.4
1.2
1.0
0.8
0.6
0.4
0.2
0.0
n.s
**
*
WT BaDV
PKI MT1
PKII MT1
PKII MT2
PKII COMP
PKIII MT1
PKIII MT2
L1.1A
L1.1B
SLIV
SLV
QC1
QC2
QC3
QC4
QC5
QC6

Chen, Chien et al_Figure S2

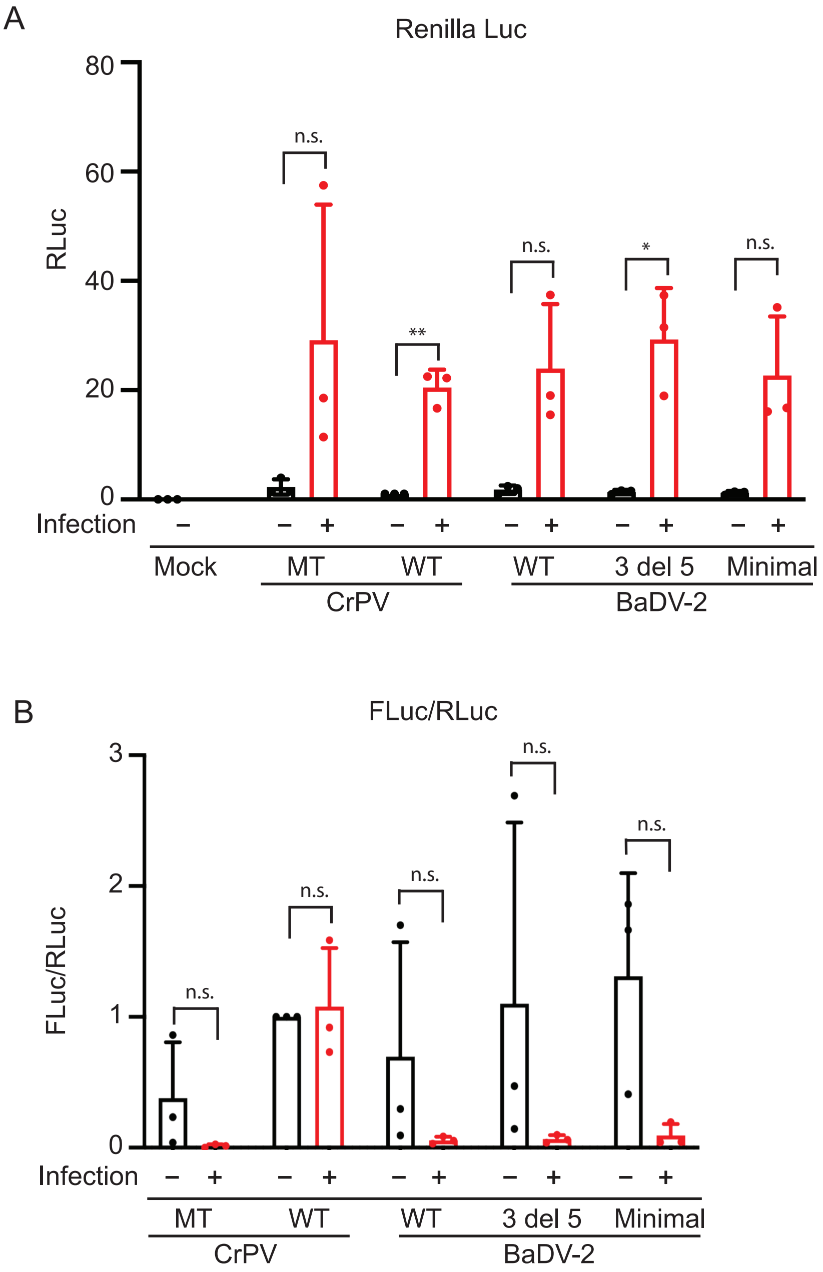

Chen et al_Figure S5

Chen et al_Figure S3

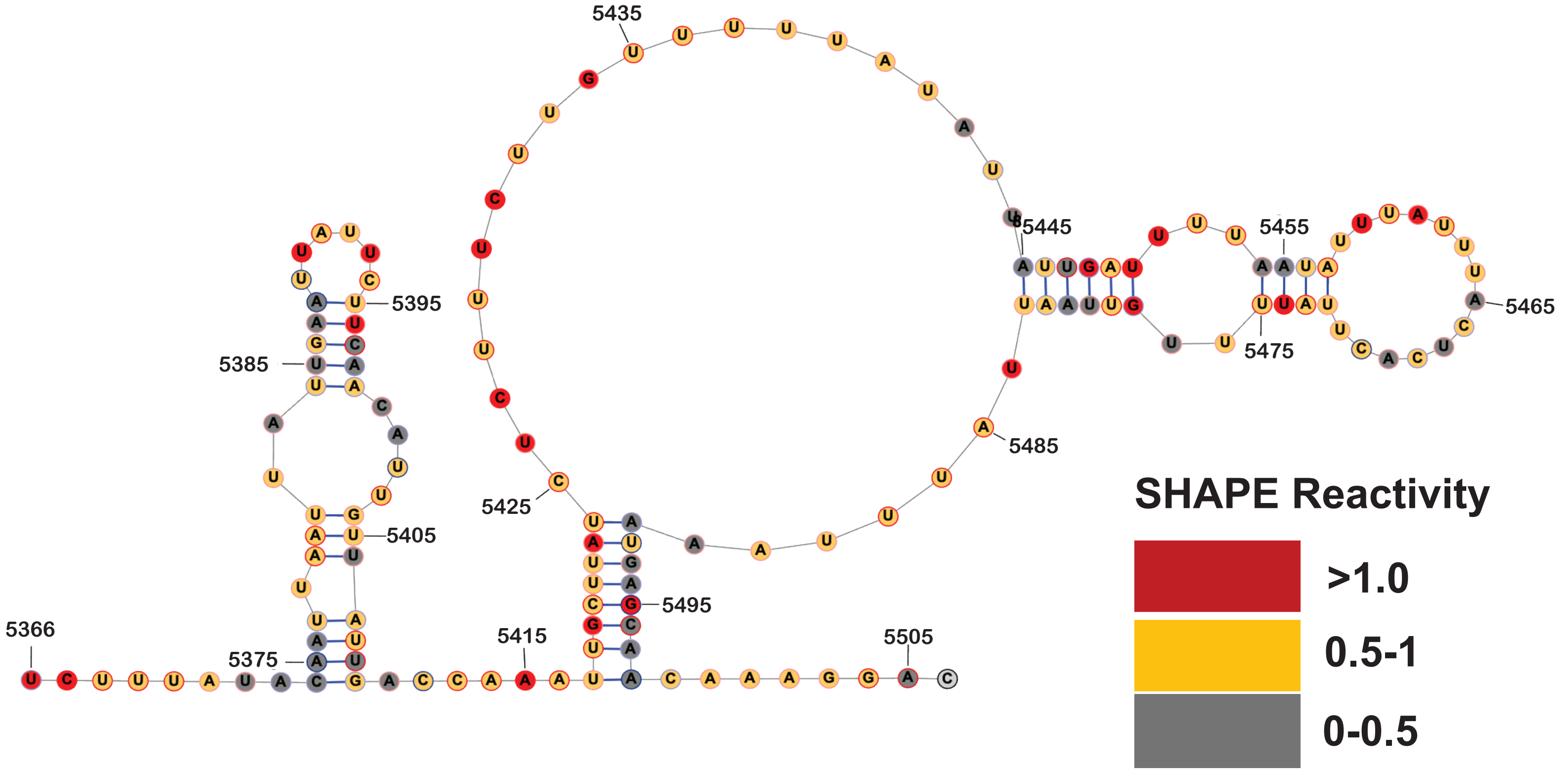

Supplement: Supplementary file 1 [file viruses-16-00695-s001.zip › viruses-2881926-supplementary.pdf]
